# Interventions to treat methicillin-susceptible *Staphylococcus aureus* bacteremia: many methodological concerns

**DOI:** 10.1186/s12879-019-4520-3

**Published:** 2019-10-25

**Authors:** Herney Andrés García-Perdomo, Jessica Fernanda Toro Maldonado

**Affiliations:** 1 Department of Surgery/Urology, School of Medicine, Universidad del Valle, Cali, Colombia; 2 Pediatric Infectious Diseases, Universidad del Valle, Cali, Colombia

**Keywords:** Methicillin-susceptible *Staphylococcus aureus*, Bacteremia, Systematic review, Meta-analysis

## Abstract

The aim of this letter was to point out some methodological concerns about an article written by Shi et al. and published in the journal. There is an increasing trend in the isolation of Methicillin-susceptible *Staphylococcus aureus* bacteremia and a variety of questions regarding the best therapy to treat this condition. These concerns might lead to selection, publication and information bias that prevent the generalization and application of these results in our clinical practice.

## Main text

Nowadays, there is an increasing trend in the isolation of Methicillin-susceptible *Staphylococcus aureus* (MSSA) bacteremia and plenty of questions regarding the best therapy to treat this kind of condition [1, 2]. According to this, we have carefully read the very recent article written by Shi et al. [3] which tries to respond the previous question, however we have found different flaws that prevent the generalization of these results.

The following are a few issues to be considered: 1. The search strategy is not presented in reproducible way, as it is described in Cochrane manual and other methodological strategies [4]. Besides, three elements are the most important to construct it: Population, Intervention and type of study [5]. On the contrary, the items shown were: Population, intervention, control and outcome, which might explain the limited number of articles found; 2. There are different strategies to saturate information in systematic reviews, however these were not shown here (P.e. Grey literature databases, clinical trial registries, thesis databases, google scholar, among others); 3.Regarding the Sensitivity analysis, authors explain that they excluded each publication, nonetheless they do not report the new results excluding the most weighted study (McDanel 2017; 81.5%) in the general analysis.

As we previously said, these important flaws, might lead to selection, publication and information bias that prevent the generalization and application of these results. Therefore, we might take them very carefully in our clinical practice.

## Supplementary information

**Additional file 1: Table S1.** Search strategy used in PubMed database. **Table S2.** Search strategy used in EMBASE database. **Table S3.** Search strategy used in the Cochrane Library database.

## Data Availability

NA. There is no data available.

## Abbreviations

MSSA: Methicillin-susceptible *Staphylococcus aureus*
