# Supplementary material for: Interventions to treat methicillin-susceptible Staphylococcus aureus bacteremia: many methodological concerns
Source: BMC Infect Dis. 2019 Oct 25;19:892. doi: 10.1186/s12879-019-4520-3 (PMC6815029; doi:10.1186/s12879-019-4520-3)
Supplement: Supplementary file 1 — Additional file 1: Table S1. Search strategy used in PubMed database. Table S2. Search strategy used in EMBASE database. Table S3. Search strategy used in the Cochrane Library database. [file 12879_2019_4520_MOESM1_ESM.docx]

Table S1. Search strategy used in PubMed database.

| Search number | Query | Items found |
| --- | --- | --- |
| #1 | (oxacillin) OR oxacillin[MeSH Terms] | 7511 |
| #2 | (nafcillin) OR nafcillin[MeSH Terms] | 929 |
| #3 | (methicillin) OR methicillin[MeSH Terms] | 34567 |
| #4 | (cloxacillin) OR cloxacillin[MeSH Terms] | 3553 |
| #5 | (floxacillin) OR floxacillin[MeSH Terms] | 684 |
| #6 | (dicloxacillin) OR dicloxacillin[MeSH Terms] | 889 |
| #7 | Flucloxacillin | 1058 |
| #8 | antistaphylococcal penicillin | 258 |
| #9 | semisynthetic penicillin | 802 |
| #10 | #1 OR #2 OR #3 OR #4 OR #5 OR #6 OR #7 OR #8 OR #9 | 42049 |
| #11 | methicillin-susceptible Staphylococcus aureus | 2688 |
| #12 | methicillin susceptible Staphylococcus aureus | 4836 |
| #13 | MSSA | 2764 |
| #14 | #11 OR #12 OR #13 | 6141 |
| #15 | (bacteremia) OR bacteremia[MeSH Terms] | 41098 |
| #16 | bacteraemia | 41098 |
| #17 | bloodstream infection | 17710 |
| #18 | (sepsis) OR sepsis[MeSH Terms] | 159862 |
| #19 | #15 OR #16 OR #17 OR #18 | 178136 |
| #20 | (cefazolin) OR cefazolin[MeSH Terms] | 4787 |
| #21 | #10 AND #14 AND #19 AND #20 | 42 |

Filters: Publication date to 2018/02/01

Table S2. Search strategy used in EMBASE database.

| Search number | Query | Items found |
| --- | --- | --- |
| #1 | 'oxacillin'/exp OR 'oxacillin' | 18300 |
| #2 | 'nafcillin'/exp OR 'nafcillin') | 5073 |
| #3 | ('methicillin'/exp OR 'methicillin') | 62239 |
| #4 | ('cloxacillin'/exp OR 'cloxacillin') | 10686 |
| #5 | ('floxacillin'/exp OR 'floxacillin') | 7655 |
| #6 | ('dicloxacillin'/exp OR 'dicloxacillin') | 4188 |
| #7 | ('flucloxacillin'/exp OR 'flucloxacillin') | 7757 |
| #8 | 'antistaphylococcal penicillin' NOT | 30 |
| #9 | 'semisynthetic penicillin' | 290 |
| #10 | #1 OR #2 OR #3 OR #4 OR #5 OR #6 OR #7 OR #8 OR #9 | 91052 |
| #11 | 'methicillin susceptible staphylococcus aureus'/exp OR 'methicillin susceptible staphylococcus aureus' | 5267 |
| #12 | 'methicillin-susceptible staphylococcus aureus'/exp OR 'methicillin-susceptible staphylococcus aureus' | 5267 |
| #13 | 'mssa'/exp OR 'mssa' | 6965 |
| #14 | #11 OR #12 OR #13 | 7377 |
| #15 | 'bacteremia'/exp OR 'bacteremia' | 50971 |
| #16 | 'bacteraemia'/exp OR 'bacteraemia' | 44603 |
| #17 | 'bloodstream infection'/exp OR 'bloodstream infection' | 12055 |
| #18 | 'sepsis'/exp OR 'sepsis' | 259194 |
| #19 | #15 OR #16 OR #17 OR #18 | 273760 |
| #20 | 'cefazolin'/exp OR 'cefazolin' | 24917 |
| #21 | #10 AND #14 AND #19 AND #20 | 182 |

Records added to Embase from: -01/02/2018

Table S3. Search strategy used in the Cochrane Library database.

| Search number | Query | Items found |
| --- | --- | --- |
| #1 | oxacillin | 214 |
| #2 | nafcillin | 76 |
| #3 | methicillin | 1288 |
| #4 | cloxacillin | 266 |
| #5 | floxacillin | 81 |
| #6 | dicloxacillin | 102 |
| #7 | flucloxacillin | 222 |
| #8 | antistaphylococcal penicillin | 16 |
| #9 | semisynthetic penicillin | 38 |
| #10 | #1 OR #2 OR #3 OR #4 OR #5 OR #6 OR #7 OR #8 OR #9 | 1903 |
| #11 | methicillin-susceptible Staphylococcus aureus | 144 |
| #12 | methicillin susceptible Staphylococcus aureus | 215 |
| #13 | MSSA | 91 |
| #14 | #11 OR #12 OR #13 | 244 |
| #15 | bacteremia | 1879 |
| #16 | bacteraemia | 679 |
| #17 | bloodstream infection | 870 |
| #18 | sepsis | 8801 |
| #19 | #15 OR #16 OR #17 OR #18 | 10977 |
| #20 | cefazolin | 1034 |
| #21 | #10 AND #14 AND #19 AND #20 | 13 |

Filters: Issue 2 of 12, February 2018
