# Supplementary material for: Interventions to treat methicillin-susceptible Staphylococcus aureus bacteremia: many methodological concerns
Source: BMC Infect Dis. 2019 Oct 25;19:892. doi: 10.1186/s12879-019-4520-3 (PMC6815029; doi:10.1186/s12879-019-4520-3)
Supplement: Related texts

# Reply: Response to: “Interventions to treat Methicillin-susceptible Staphylococcus aureus bacteremia: Many methodological concerns”

**Authors:** Changcheng Shi, Fei Teng, Nengming Lin

**Affiliations:** 3 0000 0004 1759 700X grid.13402.34 Department of Clinical Pharmacy, Affiliated Hangzhou First People’s Hospital, Zhejiang University School of Medicine, Hangzhou, China; 4 0000 0000 8744 8924 grid.268505.c College of Pharmaceutical Sciences, Zhejiang Chinese Medical University, Hangzhou, China; 5 0000 0000 9255 8984 grid.89957.3a Department of Clinical Pharmacy, Hangzhou First People’s Hospital, Nanjing Medical University, Hangzhou, China; 6 0000 0004 1759 700X grid.13402.34 Department of Clinical Pharmacology, Translational Medicine Research Center, Affiliated Hangzhou First People’s Hospital, Zhejiang University School of Medicine, Hangzhou, China

Changcheng Shi and Fei Teng contributed equally to this work

We thank Garcia-Perdomo HA and Maldonado JFT for their interest in our work [3]. The responds to the comments are as following:

1. We consider that the following three elements are required to ensure the repeatability of the search strategy: a) lists of databases, b) full search terms, and c) limitations on publication date. These three elements have been presented in our meta-analysis. Therefore, the search results of our meta-analysis are reproducible, to some extent. We agree that putting the full search strategies for each database into an Appendix may be better, as it is recommended by Cochrane Collaboration [4]. The full search strategies are described in Additional file 1: Tables S1-S3.
  We agree that it is usually unnecessary to search on every aspect of the review’s clinical question (often referred to as PICO – that is Population, Intervention, Comparison and Outcome). In our meta-analysis, three elements (Population, Intervention and Comparison) were used as the search terms but not all the elements of PICO as Garcia-Perdomo HA and Maldonado JFT pointed out. Considering the Outcome may not be well described and are often not well indexed with controlled vocabulary terms, we did not use the search terms relating to Outcome. Types of study is important (typically a ‘filter’ for randomized trials) [4]. However, both randomized trials and observational studies met the criteria of our meta-analysis. Therefore, we did not use the terms relating to type of study.
2. Regarding the database, it is recommended by Cochrane Collaboration that CENTRAL and MEDLINE should be searched, as a minimum, together with EMBASE if it is available to the review author [4]. CENTRAL is published as part of The Cochrane Library. PubMed provides access to a free version of MEDLINE that also includes up-to-date citations not have been indexed for MEDLINE yet. Therefore, we think our choice of database (PubMed, EMBASE and the Cochrane Library) is acceptable. We agree that searching more databases may be beneficial, but the other factors (e.g. time spending and budget considerations) need to be balance.
3. We have performed a sensitivity analysis to evaluate the stability of our meta-analysis by excluding the study by McDanel et al. [6] and no inconsistency results were revealed (OR, 0.56; 95% CI, 0.37 to 0.84; I^2^ = 1.6%). In fact, the data have been shown in our publication (Additional file 1: Table S3) [3].
  We agree that meta-analysis require a thorough, objective and reproducible search of a range of sources to identify as many relevant studies as possible to minimize bias.
  Once again, thank Garcia-Perdomo HA and Maldonado JFT for their comments and suggestions.
